# Effect of Parkinson’s Disease on Cardio-postural Coupling During Orthostatic Challenge

**DOI:** 10.3389/fphys.2022.863877

**Published:** 2022-06-03

**Authors:** Rabie Fadil, Asenath X. A. Huether, Ajay K. Verma, Robert Brunnemer, Andrew P. Blaber, Jau-Shin Lou, Kouhyar Tavakolian

**Affiliations:** ^1^ Biomedical Engineering Program, University of North Dakota, Grand Forks, ND, United States; ^2^ Parkinson Disease Research Laboratory, Department of Neurology, Sanford Health, Fargo, ND, United States; ^3^ Department of Biomedical Physiology and Kinesiology, Simon Fraser University, Burnaby, BC, Canada; ^4^ School of Medicine and Health Sciences, Department of Neurology, University of North Dakota, Grand Forks, ND, United States

**Keywords:** Parkinson’s disease, falls, muscle-pump, cardiac baroreflex, blood pressure regulation, cardio-postural coupling

## Abstract

Cardiac baroreflex and leg muscles activation are two important mechanisms for blood pressure regulation, failure of which could result in syncope and falls. Parkinson’s disease is known to be associated with cardiac baroreflex impairment and skeletal muscle dysfunction contributing to falls. However, the mechanical effect of leg muscles contractions on blood pressure (muscle-pump) and the baroreflex-like responses of leg muscles to blood pressure changes is yet to be comprehensively investigated. In this study, we examined the involvement of the cardiac baroreflex and this hypothesized reflex muscle-pump function (cardio-postural coupling) to maintain blood pressure in Parkinson’s patients and healthy controls during an orthostatic challenge induced *via* a head-up tilt test. We also studied the mechanical effect of the heart and leg muscles contractions on blood pressure. We recorded electrocardiogram, blood pressure and electromyogram from 21 patients with Parkinson’s disease and 18 age-matched healthy controls during supine, head-up tilt at 70°, and standing positions with eyes open. The interaction and bidirectional causalities between the cardiovascular and musculoskeletal signals were studied using wavelet transform coherence and convergent cross mapping techniques, respectively. Parkinson’s patients displayed an impaired cardiac baroreflex and a reduced mechanical effect of the heart on blood pressure during supine, tilt and standing positions. However, the effectiveness of the cardiac baroreflex decreased in both Parkinson’s patients and healthy controls during standing as compared to supine. In addition, Parkinson’s patients demonstrated cardio-postural coupling impairment along with a mechanical muscle pump dysfunction which both could lead to dizziness and falls. Moreover, the cardiac baroreflex had a limited effect on blood pressure during standing while lower limb muscles continued to contract and maintain blood pressure *via* the muscle-pump mechanism. The study findings highlighted altered bidirectional coupling between heart rate and blood pressure, as well as between muscle activity and blood pressure in Parkinson’s disease. The outcomes of this study could assist in the development of appropriate physical exercise programs to reduce falls in Parkinson’s disease by monitoring the cardiac baroreflex and cardio-postural coupling effect on maintaining blood pressure.

## Introduction

Parkinson’s disease (PD) is a neurodegenerative disorder characterized by tremors, bradykinesia, rigidity, and postural instability (Mazzoni et al., 2012). When transitioning from supine to stand or sitting to stand, a transient decrease in blood pressure occurs due to the sudden change in the hydrostatic pressure gradient between the feet and the heart. In healthy persons, homeostatic mechanisms quickly compensate. However, people with Parkinson’s disease may experience impaired reflexes and the drop in blood pressure persists (orthostatic hypotension, OH) (Fanciulli et al., 2020), leading to postural instability, visual disturbances, and loss of consciousness (Mar and Raj 2018), all of which contribute to falls in Parkinson’s disease (Allen et al., 2013). Falls can lead to injury, decreased activity due to fear of falling, poor quality of life, and caregiver stress (LeWitt et al., 2019). Studies have shown that OH can happen in the early stages of the disease and may occur with or without symptoms (Palma et al., 2015; Cutsforth-Gregory and Low 2019; Yoo et al., 2021). In this study, we investigated the involvement of the cardiac baroreflex and cardio-postural coupling (hypothesized reflex muscle-pump function) in maintaining blood pressure in Parkinson’s patients and healthy controls during an orthostatic challenge induced *via* a head-up tilt test. We also studied the mechanical effect of the heart and leg muscles contractions on blood pressure.

Preventing falls through maintenance of blood pressure and postural stability during standing depends on a series of reflex mechanisms, including the cardiac baroreflex, which is initiated by the autonomic nervous system *via* efferent neural pathways causing an elevation in heart rate, systemic vascular resistance, and cardiac contractility (Armstrong et al., 2021). Parkinson’s disease can result in damage to the nerve endings that secrete norepinephrine, the main neurotransmitter of the postganglionic sympathetic neurons (Ziemssen and Reichmann 2010; Espay et al., 2014), making blood pressure regulation challenging while standing. Norepinephrine deficiency resulting from sympathetic nerve degeneration contributes to reduced cardiac contractility and the inability to increase vascular resistance *via* sympathetic outflow (Foulon and De Backer 2018). In addition, previous research suggested that patients with PD have cardiac parasympathetic impairment along with sympathetic denervation (Awerbuch and Sandyk 1992; Shibata et al., 2009; Kim et al., 2014). Parkinson’s disease has been associated with autonomic dysfunction (Visser et al., 2004; Goldstein 2006; Velseboer et al., 2011; Goldstein 2014), and cardiovascular autonomic control deficiencies have been suggested as possible underlying reasons for the drop in blood pressure upon standing (Barbic et al., 2007). PD affects not only the autonomic control of blood pressure but also skeletal muscle function (Inkster et al., 2003; Lavin et al., 2020). During standing, skeletal muscles contract and compress the underlying veins, which leads to the pumping of venous blood pooled in the lower limbs back to the heart (skeletal muscle pump), increasing venous return and blood pressure (Verma et al., 2017; Verma et al., 2019). Xu et al. (2017, 2020), and Verma et al. (2019) found that leg muscles were activated in response to variations in blood pressure, thus preventing blood pooling, and maintaining blood pressure. They referred to this mechanism as the “muscle-pump baroreflex” (Xu et al., 2017; Verma et al., 2019; Xu et al., 2020). Therefore, blood pressure regulation during standing involves feedback from cardiovascular, and musculoskeletal systems. Due to the involvement of multiple physiological systems in the regulation of blood pressure, assessment of blood pressure exclusively through cardiovascular control may be inadequate. In addition to cardiovascular control, measurements of the musculoskeletal system can provide supplemental information on an individual’s ability to maintain blood pressure during an orthostatic challenge.

Previous research has been limited to independently studying cardiovascular or postural control of blood pressure in Parkinson’s disease (Pérez et al., 2015; Miyasato et al., 2018; Kamieniarz et al., 2021). However, evidence of the interplay between these control systems in young and older adults suggests that they should not be studied in isolation (Verma et al., 2017; Verma et al., 2019). Our previous work assessed the physiological interactions between the cardiovascular, postural, and musculoskeletal systems in young and older adults (Blaber et al., 2009; Verma et al., 2017; Xu et al., 2017; Verma et al., 2019). However, the relationship between these systems for blood pressure regulation and postural control in PD patients has yet to be comprehensively investigated. In this regard, our work strives to fill in the gaps of previous Parkinson’s research by monitoring cardiac and muscle pump baroreflexes and their role in blood pressure regulation and postural control in Parkinson’s disease. The outcomes of this work will provide insights in designing appropriate physical exercise programs that can increase the ability of leg muscles to generate force and improve muscle strength to maintain blood pressure and postural stability during standing, hence reducing unexpected falls and associated injuries.

Blood pressure, heart rate, and muscle activity variations are generated by complex control systems. It is consequently important to understand the behavior of these systems, such as trends, periodicities, as well as the coherence between the representative signals of such systems. To characterize these systems and assess their dynamic change, methods based on Fourier analysis have been developed. However, these methods are often based on the assumption that changes in cardiovascular and postural hemodynamics are stationary by assuming the statistical properties of these systems do not change with time (Tian et al., 2016). Hence, more appropriate analysis methods are required to characterize the natural non-stationary aspects of the cardiovascular and postural control systems. Continuous wavelet transform (CWT) has been used to analyze physiological signals in time-frequency space (Garg et al., 2013; Tian et al., 2016; de Boer and Karemaker 2019; Verma et al., 2019). While CWT is a common tool for analyzing localized oscillations in a time series, wavelet transform coherence (WTC) can be used to study the existence and strength of the coherence between two time series in the time-frequency domain.

This work aimed to investigate the involvement of the cardiac baroreflex and a hypothesized reflex muscle-pump function (cardio-postural coupling) in maintaining blood pressure in Parkinson’s patients and healthy controls during an orthostatic challenge. The mechanical effect of the heart and leg muscles contractions on blood pressure was also examined. We recorded EMG activity from the medial and lateral gastrocnemius (MG, LG), tibialis anterior (TA), and soleus (SOL) muscles. Wavelet transform coherence (WTC) was utilized to study the interdependency between the respective signals of the cardiovascular and musculoskeletal systems (Xu et al., 2017; Verma et al., 2019). Convergent cross-mapping (CCM) was used to calculate the degree and the direction of information flow (causality) between the representative signals of cardiovascular and musculoskeletal systems (Verma et al., 2017; Verma et al., 2019; Xu et al., 2020).

We hypothesized that: 1) The cardio-postural coupling is impaired in Parkinson’s disease along with inefficient lower limb muscles contractions to maintain blood pressure. 2) The cardiac baroreflex effect on blood pressure control is reduced during standing as leg muscles contractions also contribute to increased cardiac output and blood pressure.

The study’s findings are expected to reveal the effect of Parkinson’s disease on cardiac baroreflex and cardio-postural coupling, both of which are important mechanisms to maintain blood pressure and ensure a stable upright stance.

## Materials and Methods

### Experimental Protocol

We recruited PD patients from the Movement Disorders Clinic at Sanford Health in Fargo, ND. All PD patients met at least two of the four diagnostic criteria for PD: bradykinesia, tremor, rigidity, and postural instability, and were DOPA responders. We also recruited healthy controls from the community. PD patients and healthy controls were excluded if they had severe neurological or medical conditions, such as multiple sclerosis, stroke, epilepsy, COPD, congestive heart failure, or renal failure. Participants were also excluded if they had any implanted devices (e.g., deep brain stimulation, pacemaker). We excluded PD patients if they had a Montreal Cognitive Assessment [MOCA (Nasreddine et al., 2005)] score <21. Healthy controls were excluded if they met the criteria for mild cognitive impairment (MOCA <26). The IRB at Sanford Health and the University of North Dakota approved the protocol (IRB #1445). We obtained written informed consent from all participants. The study was conducted following the declaration of Helsinki.

All PD patients were administered the Movement Disorder Society–Unified Parkinson’s Disease Rating Scale (MDS–UPDRS) Part III (Goetz et al., 2007) to assess motor symptom severity. Participants maintained their regular schedule for PD medications. We calculated the levodopa equivalent daily dosage (LEDD) using the conversion formula proposed by (Tomlinson et al., 2010) (e.g., Sinemet: 1 *600 mg L-dopa = 600 LEDD; Selegiline 10 *10 mg = 100 LEDD). To evaluate general autonomic dysfunction, we administered the Scales for Outcomes in Parkinson’s disease–Autonomic Dysfunction [SCOPA–AUT (Visser et al., 2004)]. We measured symptom severity related to low blood pressure using the first 6-items of the Orthostatic Hypotension Questionnaire [OHQ (Kaufmann et al., 2012)]. To evaluate fatigue symptoms, we administered the Multidimensional Fatigue Inventory [MFI (Smets et al., 1995)]. The sample consisted of 24 individuals diagnosed with idiopathic PD by a neurologist and 22 healthy controls. Three PD patients were excluded, one due to a low MOCA score and the others due to noisy data. Four healthy controls were excluded due to a low MOCA score.

The protocol required participants to lie supine on a tilt table for 5 min of baseline recording. After 5 min, the table was tilted to 70° for 15 min to induce orthostatic challenge. After which, participants were asked to step off the tilt table into a force platform, where they stood upright with eyes open for 6 min. The procedure was conducted in a reduced sensory environment. The experiment was terminated immediately if the participant showed signs of dizziness, nausea, discomfort, risk of syncope (rapid and drastic drop in BP), or upon request. No participants requested early termination nor demonstrated adverse symptoms requiring withdrawal from the study.

### Data Acquisition

We recorded electrocardiogram (ECG) in the lead I electrode configuration from BIOPAC systems, continuous non-invasive blood pressure (BP) using a finger photoplethysmography cuff (Finapres Nova, FMS, Netherlands), and electromyogram (BIOPAC EMG system) from 21 PD patients (males: 13; females: 8) and 18 healthy controls (males: 4; females: 14) in supine, head-up tilt at 70° and standing positions with eyes open. The electromyogram (EMG) of four different leg muscles on each leg, bilateral tibialis anterior (TA), lateral and medial gastrocnemius (LG, MG), and soleus (SOL) were acquired following the SENIAM recommendations for sensor placement (Hermens et al., 1999). Data were acquired at a sampling rate of 2,000 Hz through BIOPAC Systems.

### Data Processing

Due to the presence of movement artifacts during the transition phase between tilt and standing, the first minute of data was not included in the analyses. Therefore, minutes two through six of supine, tilt, and standing data were analyzed. RR intervals were obtained from the ECG signal. Beat-to-beat systolic blood pressure (SBP) and diastolic blood pressure (DBP) were calculated as the maximum and the minimum value in the blood pressure waveform within a heartbeat. Mean arterial pressure (MAP) was obtained from SBP and DBP as: MAP=(SBP+2*DBP)/3 .

In this study, we were interested in investigating blood pressure control through lower limb muscles contraction, thus EMG signals from all individual muscles were added to represent the overall muscle activity (Garg et al., 2013; Garg et al., 2014; Xu et al., 2020). EMG data was rectified (absolute value), transformed to have zero-mean, and low pass filtered (cutoff frequency of 20 Hz). The EMG envelope was then captured by a moving average filter whose cutoff frequency was recommended by the SENIAM project to be within 5–20 Hz (Hermens et al., 1999). Since the cardiopostural control response occurs in the low-frequency (<0.5 Hz) (Borst and Karemaker 1983; Wallin et al., 1994; Blaber et al., 2009; Xu et al., 2017), a cutoff frequency of 5 Hz was used for the filter in EMG envelope extraction. Finally, the area under the EMG envelope within each heartbeat [EMG impulse ( ${\text{ EMG}}_{\text{imp}}$ )] was calculated to represent analogously the impulse of force. Beat-to-beat physiological signals were interpolated using the spline technique and then resampled to 10 Hz before wavelet transform coherence (WTC) and causality analysis.

In this work, we used wavelet transform coherence (Grinsted et al., 2004; Garg et al., 2013; Garg et al., 2014; Tian et al., 2016) to study the interaction in time and frequency between the following signal pairs SBP→RR (cardiac baroreflex), $\mathrm{SBP\to EM}G_{\mathrm{imp}}$ (cardio-postural coupling). Three frequency bands were investigated in this study: very low frequency (VLF, 0.03–0.07 Hz), low frequency (LF, 0.07–0.15 Hz), and high frequency (HF, 0.15–0.5 Hz). We defined three wavelet analysis-derived metrics, namely, fraction time active (FTA), gain, and active gain, as follows:

1) Fraction time active (FTA) was calculated as the portion of time during which the squared cross-wavelet coherence ${\text{R}}_{\text{n}}^{2}\left(\text{s}\right)\;$ was higher than the statistical significance threshold estimated using Monte Carlo simulation (Xu et al., 2020). FTA reflects the overall degree at which the signals of each pair were correlated at each time scale.
2) The gain reflects the relative amplitude between the signals of each pair over a specified frequency range and it was calculated as the mean value of gain at each time scale over the periods where ${\text{R}}_{\text{n}}^{2}\left(\text{s}\right)$ was higher than the statistical significance threshold (Grinsted et al., 2004).
3) The active gain was calculated as (FTA × Gain) to study the effectiveness of the cardiac baroreflex and cardio-postural coupling (Xu et al., 2020).

Convergent cross-mapping (CCM) (Sugihara et al., 2012) technique was used to study the bidirectional causalities between the following signal pairs: SBP↔RR (cardiac baroreflex vs heart rate effect on blood pressure), $\text{SBP}↔{\text{ EMG}}_{\text{imp}}$ (cardio-postural coupling vs. mechanical muscle-pump effect on blood pressure). Convergent cross-mapping results presented in this paper were calculated using an embedding dimension (E) of 5, and a delay (τ) of 15 samples (1.5 s) to capture physiological alterations within periods between successive heartbeats. These parameters were determined based on the work of (Wallot and Mønster 2018). Mean values for all signals were averaged over the beat-to-beat sequence of the 5 min of data analyzed. Only gain, active gain, and FTA for the LF region are reported in this study unless noted otherwise. More details about data analysis can be found in (Verma et al., 2017; Xu et al., 2017). Please see Supplementary Appendices SA, SB for more information on wavelet coherence transform and convergent cross-mapping.

### Statistical Analysis

Since data were recorded on supine, tilt, and standing positions and not all response variables were normally distributed, we used a nonparametric Anova-type statistic (nparLD, F1-LD-F1 design) suggested by (Brunner et al., 2002). The F1-LD-F1 design refers to an experimental design with one between-subjects factor (Parkinson’s patients and healthy controls) and one within-subjects factor (supine, tilt and stand). This design is employed to study the differences between PD and HC during supine, tilt, and stand, in addition, to investigate the interaction between the two factors on the calculated response variables. To investigate the pairwise differences between supine, tilt, and standing (time main effect), we applied multiple comparisons (LD-F1 design) with Bonferroni adjustment. Wilcoxon rank-sum tests were used to study the differences between Parkinson’s patients and healthy controls (treatment main effects) in supine, tilt, and standing positions. The test results were considered significant at α=0.05 . Tabular data are reported as mean ± SD unless noted otherwise. All statistical tests were performed using R (R Development Core Team 2010).

## Results

Parkinson’s patients varied in disease severity [Hoehn and Yahr Stage I: 12 (57%), Stage II: 7 (33%), Stage III: 2 (10%)]. All patients were on either Carbidopa/Levodopa alone or Carbidopa/Levodopa with dopamine agonists or Monoamine Oxidase Type B (MAO-B) inhibitors. Table 1 shows participants’ characteristics.

**TABLE 1:** Characteristics of Parkinson’s disease patients and healthy controls considered in this study. Body mass index (BMI), Montreal Cognitive Assessment (MOCA), Scales for Outcomes in Parkinson’s disease–Autonomic Dysfunction (SCOPA–AUT), Orthostatic Hypotension Questionnaire (OHQ), Multidimensional Fatigue Inventory (MFI), Movement Disorder Society–Unified Parkinson’s Disease Rating Scale (MDS–UPDRS), Levodopa Equivalent Daily Dosage (LEDD). The table lists the Wilcoxon rank sum comparison *p*-value between Parkinson’s disease patients (PD) and healthy controls (HC).

	HC	PD	*p*-value
Age (years)	64 ± 10	65 ± 5	0.83
Weight (kg)	73.66 ± 12.06	85.90 ± 17.12	0.01
Height (cm)	165.61 ± 9.73	173.76± 9.76	0.01
BMI ( $\mathrm{kg/}m^{2}$ )	26.90 ± 3.77	28.30 ± 4.13	0.10
< 18.5	0 (0%)	1 (5%)	—
18.5–24.9	6 (33%)	3 (14%)	—
25–29.9	10 (56%)	11 (52%)	—
30–34.9	2 (11%)	6 (29%)	—
MOCA	27.78± 1.59	26.95± 1.83	0.17
SCOPA–AUT	6.78 ± 3.21.61	13.38± 7.61	0.003
OHQ	5.67 ± 7.88	10.95 ± 12.58	0.13
MFI	40.28 ± 13.12	49.19 ± 18.39	0.13
MDS–UPDRS	—	24.90 ± 9.32	—
Hoehn and Yahr scale	—	1.52 ± 0.68	—
Stage I	—	12 (57%)	—
Stage II	—	7 (33%)	—
Stage III	—	2 (10%)	—
Medication (LEDD)	—	755.38 ± 396.27	—
Carbidopa/Levodopa alone	—	11 (52%)	—
Carbidopa/Levodopa + Rasagiline	—	4 (19%)	—
Carbidopa/Levodopa + Ropinirole	—	2 (10%)	—
Carbidopa/Levodopa + Pramipexole	—	2 (10%)	—
Carbidopa/Levodopa extended-release + Rasagiline	—	1 (5%)	—
Rasagiline alone	—	1 (5%)	—

### Cardiovascular and Musculoskeletal Variables

Averaged values (over 5 min period) of cardiovascular and musculoskeletal variables are shown in Table 2. No difference in heart rate (p = 0.47) , systolic blood pressure (p = 0.75) , diastolic blood pressure (p = 0.78) , or mean arterial pressure (p = 0.95) were observed in Parkinson’s patients compared to healthy controls. However, heart rate (p< 0.001) increased in both groups from supine to tilt, tilt to standing, and during standing as compared to the supine position. SBP and MAP decreased (p< 0.04) in Parkinson’s patients during standing as compared to the supine position, while they did not change in the healthy controls. Parkinson’s patients had higher EMG activity in supine (p = 0.01) and tilt (p< 0.001) while ${\text{EMG}}_{\text{imp}}$ was higher in the PD group during tilt (p< 0.01) . EMG and ${\text{EMG}}_{\text{imp}}\;$ increased (p< 0.001) in both groups during tilt and standing positions compared to supine. In addition, EMG and ${\text{EMG}}_{\text{imp}}$ did not change in Parkinson’s patients, while it increased (p< 0.001) from tilt to standing in the healthy controls.

**TABLE 2:** Cardiovascular and musculoskeletal parameters (mean ± SD) for Parkinson’s disease patients and healthy controls during supine, tilt, and standing positions. HR: heart rate; SBP: systolic blood pressure; DBP: diastolic blood pressure; MAP: mean arterial pressure; EMG: electromyogram; ${\text{EMG}}_{\text{imp}}\;$ beat-to-beat electromyogram; CV: coefficient of variation. The Bolded values indicate a significant difference (Wilcoxon rank sum test) between Parkinson’s patients (PD) and healthy controls (HC). *, § represent a significant difference (post-hoc analysis with Bonferroni adjustment) from supine, tilt, respectively.

	Supine		Tilt		Standing	
	HC	PD	HC	PD	HC	PD
HR (bpm)	65.47 ± 7.12	68.39 ± 9.88	74.61 ± 8.54*	76.33 ± 9.57*	82.21 ± 11.81*§	83.02 ± 10.62*§
SBP (mmHg)	126.69 ± 8.70	132.43 ± 12.97	123.38 ± 16.21	129.18 ± 16.31	130.67 ± 15.29	123.63 ± 12.01*
DBP (mmHg)	83.96 ± 10.22	87.44 ± 9.46	84.97 ± 13.83	85.00 ± 7.72	87.43 ± 11.58	83.23 ± 8.70
MAP (mmHg)	98.20 ± 9.26	102.44±10.12	97.77 ± 13.83	99.72 ± 9.88	101.85 ± 11.70	96.70 ± 8.76*
EMG (μV)	17.56 ± 9.01	**25.17 ± 11.61**	51.18 ± 49.77	**107.38 ± 73.76**	113.39 ± 45.19	109.65 ± 35.43
${\text{EMG}}_{\text{imp}}\left(\mu \text{V}.\text{s}\right)$	16.22 ± 7.93	22.82 ± 11.56	43.68 ± 48.48*	85.88 ± 6.80*	85.81 ± 41.46*§	80.74 ± 28.38*
SBP CV	0.05 ± 0.01	0.04 ± 0.03	0.07 ± 0.02*	0.05 ± 0.03*	0.07 ± 0.02*	0.05 ± 0.02*
${\text{EMG}}_{\text{imp}}$ CV	0.16 ± 0.19	0.20 ± 0.13	0.33 ± 0.27*	0.21 ± 0.13	0.16 ± 0.05*§	0.19 ± 0.08

SBP coefficient of variation was lower (p= 0.01 ) in Parkinson’s patients during tilt compared to healthy controls, while it was not different between the two groups during supine and standing positions. In addition, SBP coefficient of variation increased (p<0.01 ) in both PD and healthy controls during tilt and standing positions compared to supine. No difference in ${\text{ EMG}}_{\text{imp}}$ coefficient of variation was observed between Parkinson’s patients and healthy controls. ${\text{EMG}}_{\text{imp}}$ coefficient of variation did not change in Parkinson’s patients, while it increased (p<0.01 ) in healthy controls in tilt and standing positions compared to supine and decreased (p=0.04 ) from tilt to standing.

### Cardiac Baroreflex

**FIGURE 1:**
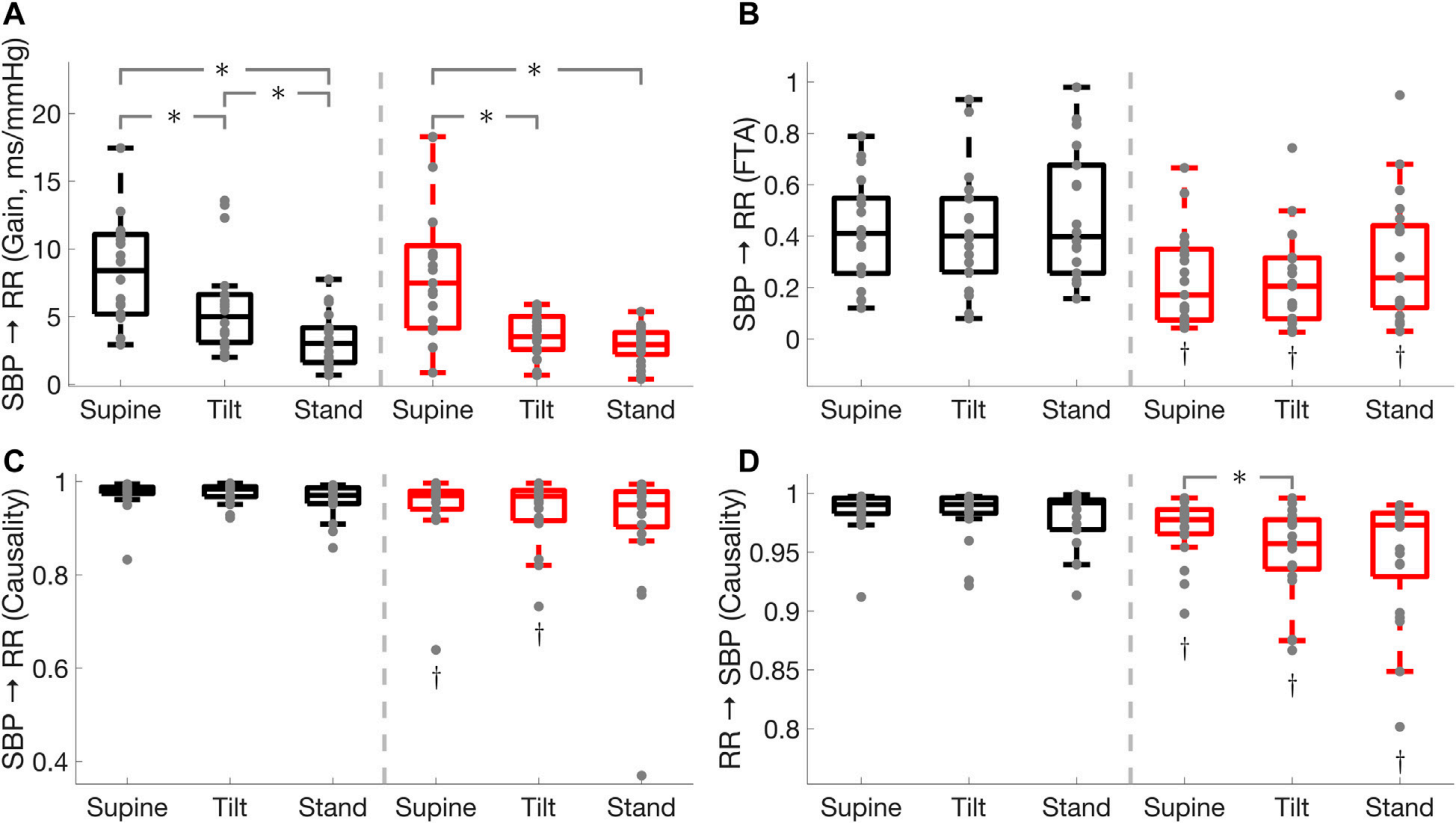
Comparison of cardiac baroreflex (SBP→RR) and the effect of heart rate on blood pressure (RR → SBP) in Parkinson’s disease patients (red) and healthy controls (black) during supine, tilt and standing positions. Baroreflex gain **(A)**, fraction time active **(B)**, causality **(C)**, and the mechanical effect of the heart on blood pressure **(D)**. * illustrates a significance difference for multiple comparison (post-hoc analysis with Bonferroni adjustment) between supine, tilt, and standing in Parkinson’s disease patients and healthy controls, while † represents a significant difference (Wilcoxon rank sum test) between Parkinson’s disease patients and healthy controls.

No difference in the cardiac baroreflex (SBP→RR) gain was found between Parkinson’s patients and healthy controls in supine, tilt, and standing. However, cardiac baroreflex gain decreased in both groups from supine to tilt ( p<0.05 ) and during standing as compared to the supine position ( p<0.001 ). In addition, cardiac baroreflex gain did not change in Parkinson’s patients while it decreased (p< 0.001) in healthy controls from tilt to standing position (Table 3; Figure 1A).

**TABLE 3:** Comparison of cardiac baroreflex, and cardio-postural coupling between healthy controls and Parkinson’s disease patients during supine, tilt, and standing positions. Table lists mean ± SD of gain, active gain (AG), fraction time active (FTA), and causality values. The Bolded values indicate a significant difference (Wilcoxon rank sum test) between Parkinson’s patients (PD) and healthy controls (HC). *, § represent a significant difference (post-hoc analysis with Bonferroni adjustment) from supine, tilt, respectively.

Cardiac baroreflex and effect of heart on BP	Supine		Tilt		Standing	
	HC	PD	HC	PD	HC	PD
SBP→ RR gain	8.37 ± 3.76	8.41 ± 5.16	5.78 ± 3.71*	3.69 ± 1.57*	3.28 ± 2.01*§	2.88 ± 1.30*
SBP →RR FTA	0.42 ± 0.20	**0.23 ± 0.18**	0.42 ± 0.24	**0.23 ± 0.19**	0.48 ± 0.25	**0.29 ±0.24**
SBP → RR AG	3.58 ± 2.40	**1.85 ± 1.73**	2.16 ± 1.61*	**0.98 ± 0.94**	1.78 ± 1.61*	0.95 ± 0.93*
SBP→ RR causality	0.97 ± 0.04	**0.95 ± 0.07**	0.98 ± 0.02	**0.94 ± 0.07**	0.96 ± 0.04	0.91 ± 0.14
RR→ SBP causality	0.98 ± 0.02	**0.97 ± 0.03**	0.98 ± 0.02	0.95 ± 0.04*	0.98 ± 0.02	**0.95 ± 0.05**
**Cardio-postural coupling and muscle-pump**						
$\text{SBP}\to {\text{ EMG}}_{\text{imp}}$ gain	0.65 ± 1.14	**0.96 ± 0.65**	1.11 ± 1.29	1.97 ± 2.06*	1.61 ± 1.07*§	3.15 ± 1.83*§
$\text{SBP}\to {\text{ EMG}}_{\text{imp}}$ FTA	0.16 ± 0.15	**0.06 ± 0.05**	0.12 ± 0.13	**0.06 ± 0.06**	0.09 ± 0.09	**0.04 ± 0.04**
$\text{SBP}\to {\text{EMG}}_{\text{imp}}$ AG	0.06 ± 0.08	0.05 ± 0.05	0.08 ± 0.08	0.07±0.06	0.13 ± 0.11*	0.13 ± 0.13*
$\text{SBP}\to {\text{EMG}}_{\text{imp}}$ causality	0.94 ± 0.03	0.94 ± 0.04	0.96 ± 0.04*	**0.95 ± 0.02**	0.91 ± 0.05*§	0.91 ± 0.05*§
${\text{EMG}}_{\text{imp}}\to \text{SBP}$ causality	0.99 ± 0.02	**0.96 ± 0.07**	0.98 ± 0.02	**0.96 ± 0.03**	0.98 ± 0.03	**0.95 ± 0.04**

**FIGURE 2:**
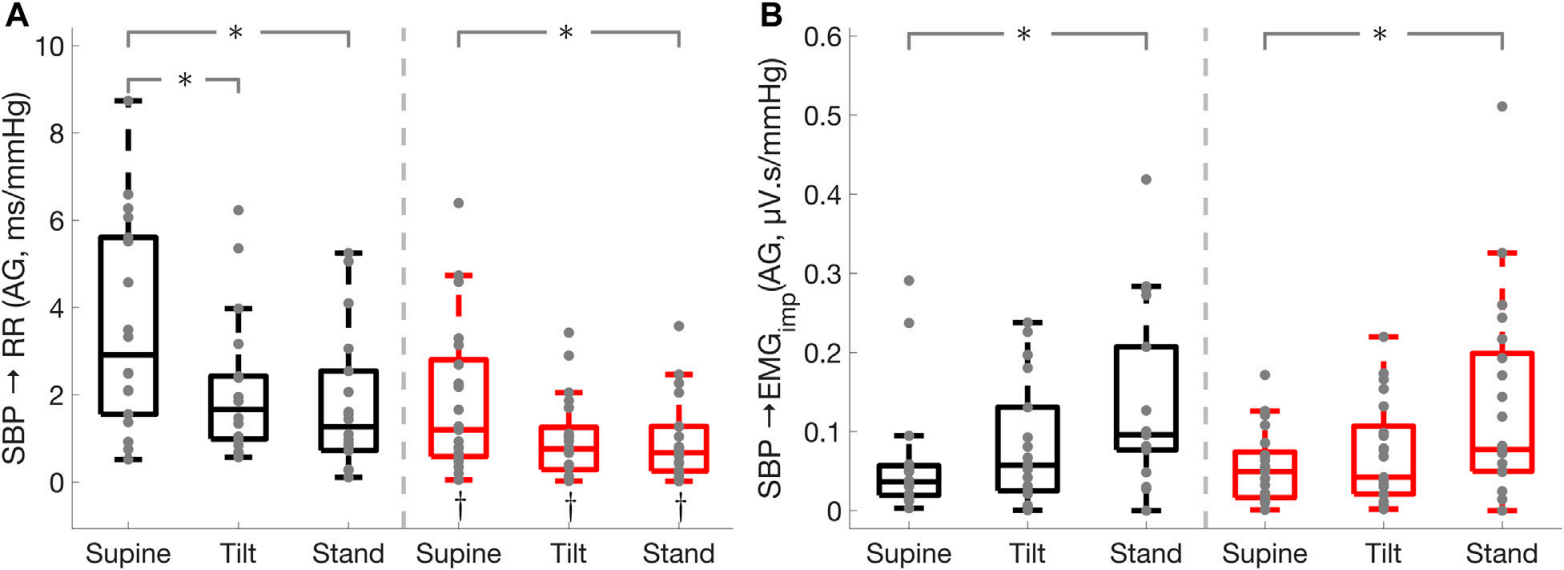
Effect of Parkinson’s disease on the effectiveness of the cardiac baroreflex and cardio-postural coupling. Cardiac baroreflex active gain **(A)**, cardio-postural coupling active gain **(B)**. * illustrates a significance difference for multiple comparison (post-hoc analysis with Bonferroni adjustment) between supine, tilt, and standing in Parkinson’s disease patients (red) and healthy controls (black), while † represents a significant difference (Wilcoxon rank sum test) between Parkinson’s disease patients and healthy controls.

**FIGURE 3:**
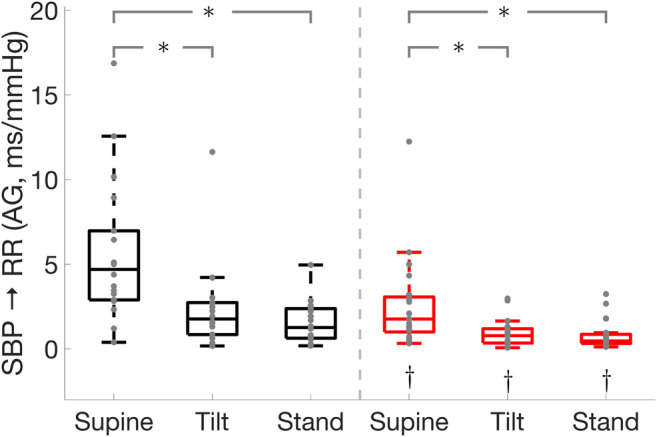
Effect of Parkinson’s disease on the cardiac baroreflex (SBP→RR) active gain in the high-frequency band. * illustrates a significance difference for multiple comparison (post-hoc analysis with Bonferroni adjustment) between supine, tilt, and standing in Parkinson’s disease patients (red) and healthy controls (black), while † represents a significant difference (Wilcoxon rank sum test) between Parkinson’s disease patients and healthy controls.

**FIGURE 4:**
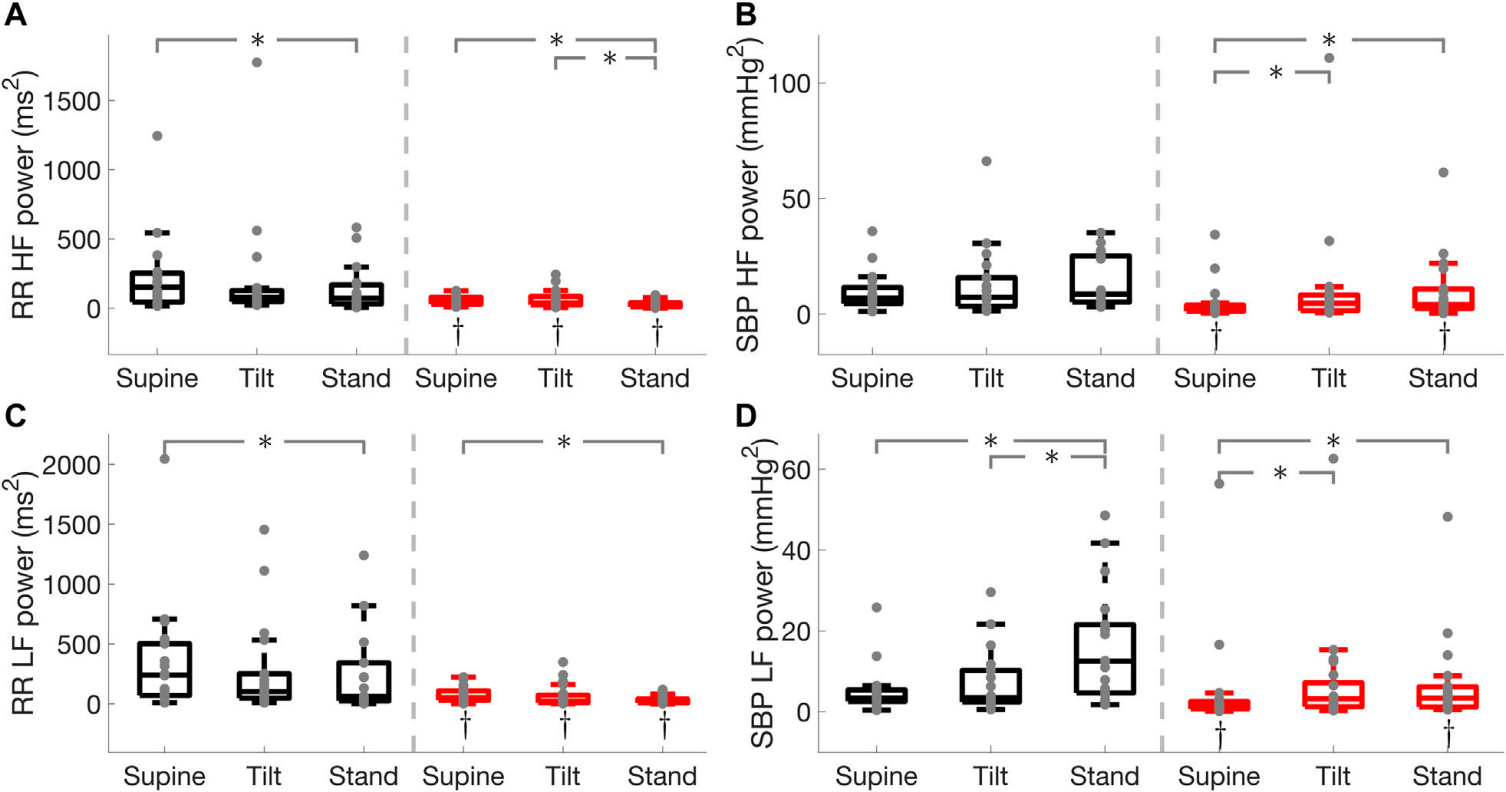
RR interval and systolic blood pressure (SBP) spectral power in the high (HF) and low (LF) frequency bands in Parkinson’s disease and healthy controls. RR interval HF power **(A)**, SBP HF power **(B)**, RR interval LF power **(C)**, SBP LF power **(D)**. * illustrates a significance difference for multiple comparison (post-hoc analysis with Bonferroni adjustment) between supine, tilt, and standing in Parkinson’s disease patients (red) and healthy controls (black), while † represents a significant difference (Wilcoxon rank sum test) between Parkinson’s disease patients and healthy controls.

**FIGURE 5:**
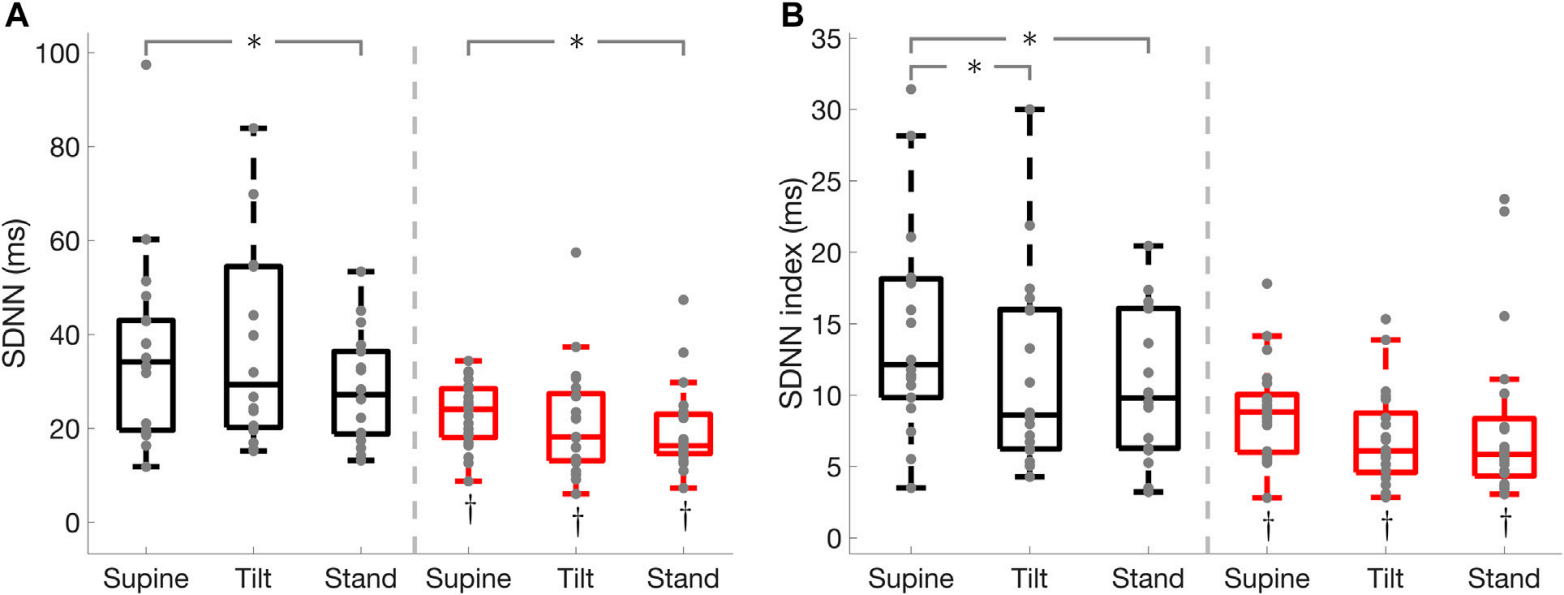
Standard deviation of normal RR interval (SDNN), and SDNN index in Parkinson’s disease and healthy controls. SDNN **(A)**, SDNN index **(B)**. * illustrates a significance difference for multiple comparison (post-hoc analysis with Bonferroni adjustment) between supine, tilt, and standing in Parkinson’s disease patients (red) and healthy controls (black), while † represents a significant difference (Wilcoxon rank sum test) between Parkinson’s disease patients and healthy controls.

Cardiac baroreflex fraction time active was lower in PD during supine ( p<0.01 ), tilt (  p<0.01 ), and standing ( p= 0.01 ) positions compared to HC (Table 3; Figure 1B), while no difference was observed between supine, tilt, and standing positions in Parkinson’s patients and healthy controls. Similarly, cardiac baroreflex active gain was lower in PD during supine ( p= 0.01 ), tilt (  p<0.01 ), and standing ( p= 0.04 ) positions as compared to HC, while it decreased (p<0.05) in both groups during standing as compared to the supine position. Moreover, cardiac baroreflex active gain did not change in Parkinson’s patients while it decreased ( p= 0.03) in healthy controls from supine to tilt position (Table 3; Figure 2A). Furthermore, the cardiac baroreflex active gain in the high-frequency band was lower in PD during supine ( p<0.01 ), tilt (  p=0.01 ), and standing ( p=0.04 ) positions as compared to HC, while it decreased (p<0.05) in both groups during tilt and standing as compared to the supine position (Figure 3). Spectral power of RR interval in the HF and LF bands, the standard deviation of normal RR interval (SDNN), and SDNN index were lower (  p<0.05 ) in PD during supine tilt and standing positions compared to HC (Figures 4A, C, Figure 5). Moreover, SBP spectral power in the HF and LF bands was lower (  p<0.05 ) in PD during supine and standing positions compared to HC (Figures 4B, D).

The cardiac baroreflex (SBP→RR) causality was lower in PD in supine (p=0.04) and tilt (p=0.01) compared to HC, while no difference was found between the groups during standing. No difference between the groups was observed during supine, tilt, and standing positions (Table 3; Figure 1C). The mechanical effect of the heart on blood pressure ( RR→SBP ) causality was lower (p<0.01) in PD in supine, tilt, and standing positions as compared to HC (Table 3; Figure 1D). Moreover, Parkinson’s patients exhibited a reduced  (p=0.01) mechanical effect of the heart on blood pressure during tilt as compared to the supine position (Table 3; Figure 1D).

### Cardio-Postural Coupling and Muscle-Pump

**FIGURE 6:**
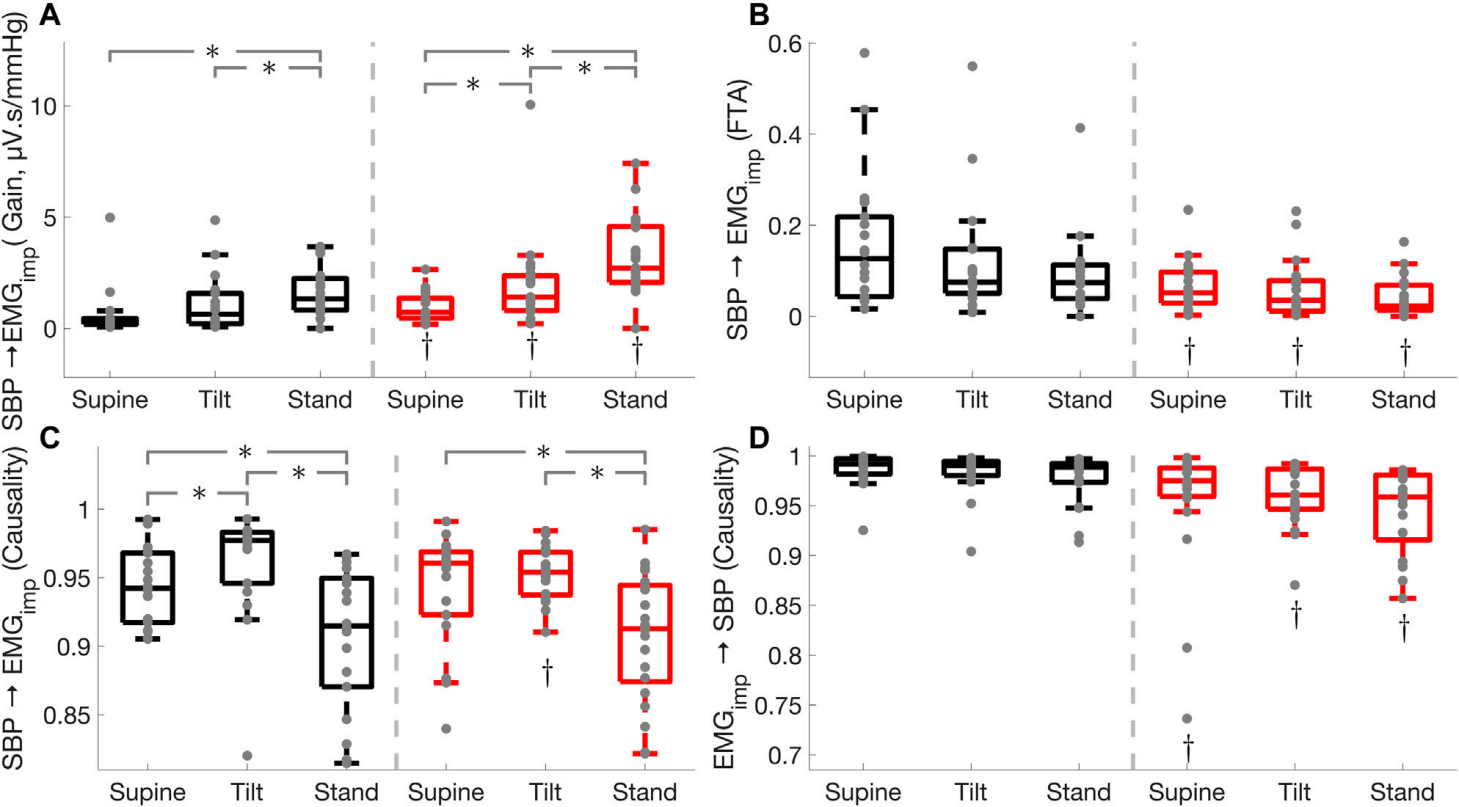
Effect of Parkinson’s disease on cardio-postural coupling ( $\text{ SBP}\to {\text{EMG}}_{\text{imp}})$ , and muscle-pump $\left({\text{EMG}}_{\text{imp}}\to \text{SBP}\right)$ . Cardio-postural coupling gain **(A)**, cardio-postural coupling fraction time active **(B)**, cardio-postural coupling causality **(C)**, and muscle-pump **(D)**. * illustrates a significance difference for multiple comparison (post-hoc analysis with Bonferroni adjustment) between supine, tilt, and standing in Parkinson’s disease patients (red) and healthy controls (black), while † represents a significant difference (Wilcoxon rank sum test) between Parkinson’s disease patients and healthy controls.

Cardio-postural coupling ( $\text{ SBP }\to {\text{ EMG}}_{\text{imp}})$ gain was higher in Parkinson’s patients during supine (p<0.01) , tilt (p=0.03), and standing (p<0.01) compared to healthy controls. In addition, cardio-postural coupling gain was increased in both groups during standing (p<0.001) and tilt (p<0.03) compared to the supine position. Furthermore, cardio-postural coupling gain was elevated (p<0.01) in Parkinson’s patients while it did not change in healthy controls when going from tilt to standing (Table 3; Figure 6A). Cardio-postural coupling active gain was increased in the standing position compared to supine (p<0.05) in both Parkinson’s patients and healthy controls, while no difference between the groups was observed during supine, tilt, and standing positions (Table 3; Figure 2B).

Cardio-postural coupling fraction time active was lower in Parkinson’s patients during supine (p=0.01) , tilt (p=0.03) , and standing (p<0.01) compared to healthy controls, while no difference was observed between supine, tilt, and standing positions in Parkinson’s patients and healthy controls (Table 3; Figure 6B). Cardio-postural coupling ( $\text{ SBP }\to {\text{EMG}}_{\text{imp}}\;)$ causality was lower in PD patients during tilt (p= 0.02) compared to healthy controls, while no difference was observed between the groups during supine and standing positions. Moreover, cardio-postural coupling causality increased from tilt to standing (p<0.001) and was higher in standing compared to the supine position (p<0.03) in both groups. In addition, cardio-postural coupling causality did not change in Parkinson’s patients while it increased ( p= 0.04) in healthy controls from supine to tilt position (Table 3; Figure 6C). Muscle-pump $\;\left({\text{ EMG}}_{\text{imp}}\;\to \text{ SBP}\right)$ was lower in Parkinson’s patients in all positions (supine: p= 0.02 , tilt: p< 0.01, standing: p< 0.01) as compared to healthy controls, while no difference was observed across supine, tilt, and standing positions in both groups (Table 3; Figure 6D).

**FIGURE 7:**
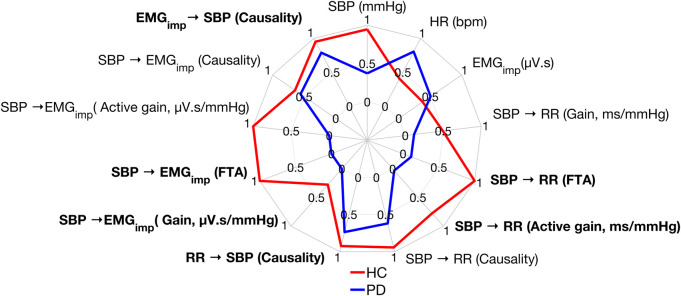
Comparison of the response variables calculated in this work between a Parkinson’s patient and a healthy control participant during standing. Data were normalized between 0 and 1 for better visualization of the results. A value of 0, 1 signifies the worst, best performance, respectively. Bolded text indicates a significant difference between Parkinson’s patients and healthy controls during standing.

Figure 7 shows the different parameters calculated in this study for one Parkinson’s patient and an age-matched healthy control participant during standing. The Parkinson’s patient presented with lower values for all the response variables compared to the healthy control participant, except heart rate, ${\text{ EMG}}_{\text{imp}}$ and $\text{ SBP }\to {\text{EMG}}_{\text{imp}}\;$ causality which are not significantly different between the groups during standing.

## Discussion

The current study assessed blood pressure regulation in Parkinson’s disease patients and healthy controls through the cardiac baroreflex and a hypothesized reflex muscle-pump function (cardio-postural coupling) during supine, head-up tilt at 70°, and standing. In addition to cardiac baroreflex dysfunction, we found that Parkinson’s disease patients experience reduced mechanical muscle pump (the mechanical effect of leg muscles contractions on blood pressure) and cardio-postural coupling (reflex activity). Impairments in these orthostatic reflexes could result in dizziness and an unexpected fall. We found that adjustments of blood pressure through leg muscles contractions during standing may compensate for, or limit the effect of, the cardiac baroreflex on blood pressure since the muscle-pump had already counteracted changes in blood pressure.

Parkinson’s disease is known to be associated with cardiac baroreflex dysfunction, however, the effect of Parkinson’s disease on cardio-postural coupling has not been previously investigated. Upon standing, 500–1,000 ml of blood shift from the central body compartments to the lower extremities and splanchnic circulation (Lanier et al., 2011), leading to a decreased venous return, cardiac output, and a consequent decline in blood pressure. The drop in blood pressure triggers reflex responses through a hypothesized cardio-postural control center, including cardiac baroreflex and the hypothesized reflex muscle-pump function. The reflex muscle-pump responds to changes in blood pressure by activating leg muscles contractions (muscle-pump) to prevent blood pooling and maintain blood pressure (Xu et al., 2017; Verma et al., 2019; Xu et al., 2020)

Muscle strength deficits and impaired cardiac baroreflex control are responsible for postural instability and contribute to falls in Parkinson’s disease (De Pablo-Fernandez et al., 2017; Gandolfi et al., 2018; Chen et al., 2020). As a result, a thorough understanding of alterations in cardiac baroreflex and cardio-postural coupling might help comprehend the physiology of blood pressure regulation in Parkinson’s disease. Such knowledge can aid in the development of appropriate measures to prevent falls in Parkinson’s disease patients.

### Cardiac Baroreflex

Parkinson’s disease patients displayed impaired cardiac baroreflex as shown by the reduced active gain, fraction time active, and RR interval spectral power in the low-frequency band which is thought to be mediated by both sympathetic and parasympathetic activities with a parasympathetic dominance (Shaffer and Ginsberg 2017). In addition, Parkinson’s patients exhibited a lower active gain and RR interval spectral power in the high-frequency band (Figure 3) which reflects parasympathetic modulation of the heart (Shaffer and Ginsberg 2017). This suggests that Parkinson’s patients have impaired sympathetic and vagal modulations. Moreover, Parkinson’s disease patients had lower SBP spectral power in the LF band which is associated with sympathetic vasomotor control (Pagani et al., 1996; Porta et al., 2012; Barbic et al., 2014). While the cardiac baroreflex was lower in Parkinson’s patients compared to healthy controls, its effectiveness was reduced in both groups during standing as compared to the supine position.

Patients with Parkinson’s disease exhibit varying degrees of autonomic dysfunction, which can occur in the early stages of the disease, and symptoms worsen as the Hoehn and Yahr PD stage progresses (Ziemssen and Reichmann 2010; Postuma et al., 2013; De Pablo-Fernandez et al., 2017). The effect of the heart on maintaining blood pressure ( RR→SBP causality) was lower in Parkinson’s group in supine, tilt and during standing compared to healthy controls, while it decreased during tilt in Parkinson’s patients as compared to supine. This suggests abnormalities in heart rate variations to maintain blood pressure in Parkinson’s disease. These alterations in heart rate variability are thought to be linked to parasympathetic system dysfunction in the early stages of Parkinson’s disease.

The cardiac baroreflex gain and active gain were reduced from supine to standing in both groups. Upon standing, lower leg muscles contract and maintain blood pressure *via* the muscle pump mechanism, and thus the effect of the cardiac baroreflex on blood pressure control was lessened in Parkinson’s patients and healthy controls during standing. This is supported by the increased leg muscle activity, muscle-pump gain, and muscle-pump active gain during standing in both groups as compared to supine position.

### Cardio-Postural Coupling and Muscle-Pump

Research have shown that the cerebellar fastigial nucleus (FN) which innervates antigravity muscles to maintain postural balance has heavy branches to the nucleus tractus solitarius (NTS), the primary integrative center for respiration and cardiovascular control (Andrezik et al., 1984; Lutherer et al., 1989; Rector et al., 2006; Zhang et al., 2016; Fujita et al., 2020). NTS receives afferent information from the arterial baroreceptors and produces autonomic adjustments to maintain blood pressure. Moreover, fastigial activity has been shown to vary as a function of arterial blood pressure (Lutherer et al., 1989; Rector et al., 2006). In addition to postural control, FN has a profound influence on cardiovascular control and respiration (Andrezik et al., 1984), while FN activity might be a tonic contribution to the autonomic response to hypotension rather than an integral part of the autonomic reflex (Chen et al., 1994). Furthermore, Andrezik et al. (1984) found that neurons that regulate posture in the rostral fastigial nucleus (rFN) are mixed in with neurons that influence the autonomic nervous system in beagles. Moreover, rFN neurons that respond to passive movement also showed changes in firing rate to cardiovascular and respiratory challenges (Lutherer et al., 1989). This suggests that rFN contributes to adaptive autonomic reflex modifications in response to changes in body posture (Rector et al., 2006; Fujita et al., 2020). However, there are certain discrepancies between investigators regarding the role of the FN area on cardiovascular and respiratory control. Some studies have suggested that the effect of the FN on cardiovascular function may result from activation of fibers of passage in autonomic control (Chida et al., 1986; Bradley et al., 1987; Miura and Takayama 1988). Moreover, NTS has been shown to have projections to the paragigantocellularis nucleus (PGi) that sends excitatory fibers to the locus coeruleus (LC) (Williams et al., 2000; Mello-Carpes and Izquierdo 2013; Lopes et al., 2016), which has been shown to modulate neuronal activity of fastigial nucleus (Somana and Walberg 1978; Tao et al., 1998; Zhang et al., 2016; Yu and Wang 2022).

Based on the above information, we suggest that the neurophysiological pathways of the hypothesized blood pressure reflex (cardio-postural coupling) could be as follows: In the supine position, gravitational forces are similar on the thorax, abdomen, and legs as these compartments lie in the same horizontal axis. Consequently, arterial and venous pressures are distributed evenly throughout the body. Upon tilting, gravity acts on the vascular volume causing blood to pool in the lower extremities leading to greater postural variations in blood pressure as compared to the supine position (Table 2). These changes in blood pressure lead to the decrease of baroreceptors discharge to the NTS which relays that information to the rFN (NTS → PGi → LC → FN) that sends efferent pathways to lower limb muscles to contract and restore arterial blood pressure.

During the head-up tilt test, participants’ feet were not in contact with any support, suggesting that the increase of muscle activity ( ${\text{EMG}}_{\text{imp}})$ during tilt as compared to the supine position (Table 2) is produced by reflex muscle-pump activity (cardio-postural coupling) as a response to blood pressure fluctuations. During tilt, cardio-postural coupling was lower in Parkinson’s patients as compared to healthy controls. Moreover, cardio-postural coupling and ${\text{EMG}}_{\text{imp}}$ coefficient of variation did not change from supine to tilt in PD, while they increased in the healthy controls. This provides support for cardio-postural coupling dysfunction in Parkinson’s patients. Such impairment, if not compensated *via* other mechanisms, could lead to dizziness and falls in Parkinson’s patients. In addition to blood pressure control, lower limb muscle activation plays an important role in maintaining balance during standing, forming a closed-loop between the cardiovascular and postural systems (cardiopostural control) (Xu et al., 2017). Therefore, during standing, leg muscles contract to maintain postural balance and blood pressure, with possible reduced cardio-postural coupling ( $\text{ SBP }\to {\text{EMG}}_{\text{imp}})$ causality observed in both groups during standing as compared to supine and tilt positions (Table 3; Figure 6D).

Parkinson’s patients exhibited higher cardio-postural coupling ( $\text{ SBP }\to {\text{EMG}}_{\text{imp}}$ ) gain in supine, tilt, and during standing compared to healthy controls (Table 3; Figure 6A). These findings might result from muscle rigidity, one of the primary motor symptoms of Parkinson’s disease, causing muscles to remain stiff and unable to relax. The increased muscle activity in Parkinson’s patients in a relaxed supine position, when compared to healthy controls, is an indicator of muscle stiffness in PD. According to previous studies, the majority of Parkinson’s patients (92%) exhibit rigidity as a motor sign of the disease (Kang et al., 2005). Muscle rigidity could result in prolonged leg muscles contractions, leading to a higher cardio-postural coupling gain in Parkinson’s patients. Contrary to cardio-postural coupling gain, cardio-postural coupling fraction time active was lower in Parkinson’s patients in supine, tilt, and during standing compared to healthy controls (Table 3; Figure 6B). This decline in the interaction between SBP and ${\text{ EMG}}_{\text{imp}}$ in terms of significant time active may result in a limited effect of the muscle pump on blood pressure in Parkinson’s patients. The reduced active time may also reflect muscle imbalance and muscle relaxation deficits in Parkinson’s disease, caused by impaired voluntary motoneuron derecruitment control (Grasso et al., 1996; Kato and Kanosue 2015; Gandolfi et al., 2018; Kato et al., 2019). A shorter significant time of interaction with blood pressure could be caused by dyssynchronous leg muscles contractions. Delayed and dyssynchronous leg muscles contractions provide support for cardio-postural coupling impairment in Parkinson’s disease. Moreover, we suggest that the hypothesized reflex muscle-pump function depends on the aortic and carotid baroreceptors input to the NTS through the vagus and glossopharyngeal nerves, therefore peripheral nervous system denervation in Parkinson’s disease (Comi et al., 2014; Walter et al., 2018; Horsager, 2021) might contribute to cardio-postural coupling dysfunction in PD. In addition, cardio-postural coupling gain and active gain increased in both groups during standing as compared to the supine position providing possible compensatory support for the reduced cardiac baroreflex effect on blood pressure during standing (Table 3; Figures 2B, 6A).

Muscle pump $\;\left({\text{ EMG}}_{\text{imp}}\;\to \text{ SBP}\right)$ was lower in Parkinson’s patients in supine, tilt, and standing positions compared to healthy controls. SBP variation increased in both groups during standing compared to the supine position (Table 2). $\;{\text{ EMG}}_{\text{imp}}\;$ variation did not change in PD during standing as compared to the supine position, while it increased in the healthy controls (Table 2). Furthermore, leg muscle activity increased in both groups during standing as compared to the supine position (Table 2). However, the translation of this increased leg muscle activity to maintain blood pressure $\;\left({\text{ EMG}}_{\text{imp}}\;\to \text{ SBP}\right)$ is lower in Parkinson’s disease compared to healthy controls (Table 3; Figure 6D). This could be due to leg muscles stiffness and dyssynchronous contractions, both of which interfere with the muscle pump function to maintain blood pressure in Parkinson’s disease. An impaired muscle pump signifies ineffective leg muscles contractions toward pumping venous blood back to the heart and increasing blood pressure in Parkinson’s disease during orthostatic challenge. Such deficit, if not compensated *via* other regulatory mechanisms, could lead to dizziness resulting in an unexpected fall. Muscle weakness is prominent in Parkinson’s disease patients (Inkster et al., 2003; Gandolfi et al., 2018), which could lead to reduced venous return, resulting in decreased cardiac output and blood pressure in Parkinson’s patients during standing (Table 3).

## Limitations and Future Work

Parkinson’s patients studied in this research were in the early stages of the disease; therefore, the role of the cardiac baroreflex and cardio-postural coupling towards blood pressure regulation and the mechanical effect of the heart and leg muscles contractions on blood pressure is still to be explored in the advanced stages of Parkinson’s disease. Parkinson’s patients in different disease stages show varying degrees of tremor and bilateral desynchronization which can significantly interfere with the muscle pump’s function to maintain blood pressure. A direct comparison of the proposed aggregated EMG marker between healthy subjects and PD patients in different stages may thus be affected from this fact. Further work will be focused on the proposal of non-aggregated EMG markers, allowing for a more detailed analysis of the muscle pump function in PD patients. Moreover, the effect of respiration on blood pressure was not investigated in this study. Respiration influences both blood pressure and postural stability (Rodrigues et al., 2018; Nuckowska et al., 2019); therefore, future studies addressing the physiology of blood pressure regulation should include respiration as well. Furthermore, the sample size of each group studied was limited; additional research with larger cohorts is needed to fully confirm the effect of Parkinson’s disease on muscle-pump and cardio-postural coupling during the orthostatic challenge. Finally, the analysis of the first minute of data was not possible, mainly due to the noise observed in the signals during this period. However, further analysis of this segment of data could provide additional information on the underlying mechanisms of the hypothesized reflex-dependent muscle activation.

## Conclusion

In this study, we investigated the involvement of the cardiac baroreflex and cardio-postural coupling—a hypothesized blood pressure reflex involving the skeletal muscle-pump–in maintaining blood pressure in Parkinson’s disease patients and healthy controls. We also studied the mechanical effect of the heart and leg muscles contractions on blood pressure. The results support the previous research suggesting that the cardiac baroreflex is impaired in PD patients and is likely driven by sympathetic and parasympathetic dysfunction. Furthermore, our study demonstrated novel findings that Parkinson’s disease patients exhibited higher cardio-postural coupling gain but lower cardio-postural coupling active time during supine, tilt, and standing. In addition, the effect of leg muscle activation to maintain blood pressure was lower in Parkinson’s patients during supine, tilt, and standing. We argue that PD-related rigidity, muscle weakness, and delayed muscle responses contribute to PD patients engaging dyssynchronous and ineffective leg muscles contractions without seeing an impactful benefit on blood pressure control. This would suggest PD patients experience a mechanical muscle pump impairment and cardio-postural coupling dysfunction which both could lead to dizziness resulting in an unexpected fall. We have also found that the cardiac baroreflex has a limited effect on blood pressure during standing as lower limb muscles contract to pump blood back to the venous circulation, increasing cardiac output and blood pressure *via* the muscle-pump mechanism. In conclusion, we found several factors that contribute to poor blood pressure regulation in PD. These factors have meaningful interaction that can contribute to dizziness, fainting, and falls in PD patients. It is imperative to recognize that the findings in this study are not limited to cardiac autonomic dysfunction alone but reflect a greater system entailing lower leg involvement in blood pressure regulation. Finally, causality analysis and wavelet-derived metrics of fraction time active (FTA), gain, and active gain for quantitative evaluation of blood pressure control in Parkinson’s disease indicate a potential to use this methodology for the development of effective interventions to reduce falls in Parkinson’s disease by monitoring the cardiac baroreflex and cardio-postural coupling and their role in maintaining blood pressure and postural stability.

## Data Availability

The datasets presented in this article are not readily available because data may only be shared for the use under which it was ethically approved. Requests to access the datasets should be directed to the corresponding author.
