# Supplementary material for: Effect of Parkinson’s Disease on Cardio-postural Coupling During Orthostatic Challenge
Source: Front Physiol. 2022 Jun 3;13:863877. doi: 10.3389/fphys.2022.863877 (PMC9214860; doi:10.3389/fphys.2022.863877)
Supplement: Supplementary file 1 [file DataSheet1.pdf]

1 Appendix A: Wavelet transform

Continuous wavelet transform (CWT) decomposes a time series in time-frequency domain by successively convolving the time series with a mother wavelet function ψ_0 which is stretched in time by varying its scale (s) and normalized to have unit energy (Torrence and Compo 1998). In this work we used the Morelet wavelet ($\omega_0 = 6$) defined as:

$$\psi_0(\eta) = \pi^{-\frac{1}{4}} e^{i\omega_0\eta} e^{-\frac{\eta^2}{2}} \tag{1}$$

The continuous wavelet transform of a time series X of length N with values x_n $(n = 1, \ldots, N)$ sampled from a continuous signal at a time step of Δt is defined as:

$$W_n^X(\mathrm{s}) = \left(\frac{\Delta t}{s}\right)^{\frac{1}{2}} \sum_{n'=1}^{N} x_{n'}\, \psi_0^* \left[(n - n')\frac{\Delta t}{s}\right] \tag{2}$$

where s is the stretch parameter used to change the scale, n is the translation parameter used to slide the wavelet function in time, and $*$ indicates the complex conjugate (Grinsted et al. 2004). Large scales correlate with the low-frequency components of the signal, while small scales are associated with the high-frequency components. In analogy to Fourier analysis, a wavelet power spectrum (Tian et al. 2016) of a time series X with values x_n can be defined as follows:

$$W_n^{XX}(s) = W_n^X(s)\, W_n^{X^*}(s) = |W_n^X(s)|^2 \tag{3}$$

Given two time series X and Y with values x_n and y_n and wavelet transforms $W_n^X(s)$ and $W_n^Y(s)$, the cross wavelet transform (XWT) of X and Y is defined as:

$$W_n^{XY}(s) = W_n^X(s)\, W_n^{Y^*}(s) \tag{4}$$

Where * denotes the complex conjugate.

The cross-wavelet power between X and Y is defined as $|W_n^{XY}(s)|$ and reveals areas with high common power, while the complex argument of $W_n^{XY}(s)$ represents the relative phase between X and Y (Grinsted et al. 2004).

The gain between two time series X and Y can be expressed as follows:

$$G_n^{XY}(s) = \left|\frac{W_n^{XY}(s)}{W_n^{XX}(s)}\right| \tag{5}$$

The squared cross-wavelet coherence $R_n^2(s)$ measures the localized correlation coefficient between two time series X and Y in the time-frequency domain and ranges between 0 and 1. The squared cross-wavelet coherence wavelet coherence is defined as follow.

$$R_n^2(s) = \frac{|\langle s^{-1} W_n^{XY}(s) \rangle|^2}{\langle s^{-1} |W_n^X(s)|^2 \rangle \langle s^{-1} |W_n^Y(s)|^2 \rangle} \quad (6)$$

where $\langle\,.\,\rangle$ is a smoothing operator in both time and scale dimensions. Smoothing is required to remove the singularities in wavelet power spectra, and enhance regions of significant power, which can be accomplished using a weighted running average in both the time and scale directions, as described by (Torrence and Compo 1998).

The statistical significance threshold of $R_n^2(s)$ can be estimated using a Monte Carlo simulation with a large ensemble of surrogate data set pairs having the same coefficients as the real input data pair based on the first-order autoregressive (AR1) model (Grinsted et al. 2004).

2 Appendix B: Convergent cross mapping

Convergent cross mapping is a technique used to calculate the bidirectional causal relationship between two time series X $(x_t, t = 1, \dots, L)$ and Y $(y_t, t = 1, \dots, L)$ where L is the length of the time series. CCM relies on state-space reconstruction to infer causality by measuring the extent to which historical values of X can be used to accurately estimate the sates of Y (cross-mapping) (Sugihara and May 1990; Sugihara et al. 2012). To do so, the lagged coordinates of variables X and Y, are used to construct the shadow manifold of $X(M_X)$ and $Y(M_Y)$ respectively. The lagged coordinates of X $(\tilde{x}_t)$ and $Y(\tilde{y}_t)$ are formed (Tsonis et al. 2018; Barraquand et al. 2021) as follows:

$$\tilde{x}_t = (x_t, x_{t-\tau}, x_{t-2\tau}, \dots, x_{t-(E-1)\tau}) \tag{7}$$

$$\tilde{y}_t = (y_t, y_{t-\tau}, y_{t-2\tau}, \dots, y_{t-(E-1)\tau}) \tag{8}$$

Where $t = 1 + (E - 1)\tau, \dots, L$, E is the embedding dimension, and τ is the time lag. Each of the vectors $\tilde{x}_t$ and $\tilde{y}_t$ represents a point in the E −dimensional space. The set of vectors $\{\tilde{x}_t\}$ and $\{\tilde{y}_t\}$ constitute the reconstructed M_X and M_Y manifolds, respectively. The next step is to find the minimum $E + 1$ nearest neighbors of each $\tilde{x}_t$ in M_X. Let's note the time indices (from closest to farthest) of the $E + 1$ nearest neighbors of $\tilde{x}_t$ by $t_1, t_2, \dots, t_{E+1}$. The nearest neighbors of $\tilde{x}_t$ in M_X are used to estimate Y as follows.

$$\hat{Y} \Big| M_X = \sum_{i=1}^{E+1} w_i \, y_{t_i} \tag{9}$$

With $w_i = u_i / \sum_{j=1}^{E+1} u_j$, $u_j = \exp\left[-d(\tilde{x}_t, \tilde{x}_{t_j}) / d(\tilde{x}_t, \tilde{x}_{t_1})\right]$, and $d(\tilde{x}_t, \tilde{x}_s)$ is the Euclidean distance between the two vectors $\tilde{x}_t$, and $\tilde{x}_s$. Predicting Y by M_X is equivalent to Y causing X, and the strength of causality flowing from Y to X is quantified by calculating the Pearson correlation coefficient between the original time series Y and the estimated $\hat{Y}|M_X$. Similarly, to know if X is causing Y (cross mapping of X by using M_Y: $\hat{X}|M_Y$), we can calculate the Pearson correlation coefficient between X and $\hat{X}|M_Y$.

3 References

Barraquand, F., Picoche, C., Detto, M. and Hartig, F. 2021. Inferring species interactions using Granger causality and convergent cross mapping. *Theoretical Ecology* 14(1), pp. 87–105. doi: 10.1007/s12080-020-00482-7.

Grinsted, A., Moore, J.C. and Jevrejeva, S. 2004. Application of the cross wavelet transform and wavelet coherence to geophysical time series. *Nonlinear Processes in Geophysics* 11(5/6), pp. 561–566. doi: 10.5194/npg-11-561-2004.

Sugihara, G., May, R., Ye, H., Hsieh, C., Deyle, E., Fogarty, M. and Munch, S. 2012. Detecting causality in complex ecosystems. *Science (New York, N.Y.)* 338(6106), pp. 496–500. doi: 10.1126/science.1227079.

Sugihara, G. and May, R.M. 1990. Nonlinear forecasting as a way of distinguishing chaos from measurement error in time series. *Nature* 344(6268), pp. 734–741. doi: 10.1038/344734a0.

Tian, F., Tarumi, T., Liu, H., Zhang, R. and Chalak, L. 2016. Wavelet coherence analysis of dynamic cerebral autoregulation in neonatal hypoxic–ischemic encephalopathy. *NeuroImage: Clinical* 11, pp. 124–132. doi: 10.1016/j.nicl.2016.01.020.

Torrence, C. and Compo, G.P. 1998. A Practical Guide to Wavelet Analysis. *Bulletin of the American Meteorological Society* 79(1), pp. 61–78. doi: 10.1175/1520-0477(1998)079<0061:APGTWA>2.0.CO;2.

Tsonis, A.A., Deyle, E.R., Ye, H. and Sugihara, G. 2018. Convergent Cross Mapping: Theory and an Example. In: Tsonis, A. A. ed. *Advances in Nonlinear Geosciences*. Cham: Springer International Publishing, pp. 587–600. Available at: https://doi.org/10.1007/978-3-319-58895-7_27.
